# 

**Published:** 1882-01

**Authors:** 


### *TABLE OF CONTENTS.*

ORIGINAL COMMUNICATIONS.

ART. I.—External Urethrotomy With Cases. By CHARLES F. BEVAN, M. D. 1

ART. 2.—Some Remarks on the Destruction of Shade Trees as a Sanitary Measure. By RUFUS W. GRISWOLD, M. D.................... 8

ART. 3.—Clinical Reports. A New Operation for Exsection of the Inferior Maxillary Nerve in the Spheno-Maxillary Fossa................ 11

ORIGINAL TRANSLATIONS AND ABSTRACTS.

The Surgery of the Pericardium.................................... 12
The Modern Treatment of Wounds.................................... 15
Vaccination with Animal and Human Virus........................... 17
Quinine in Gynecic and Obstetric Practice......................... 21
The Innervation of the Salivary Glands............................ 23

SELECTIONS.

Accidental Ante-Partum Hemorrhage................................. 24
Civilization in its Relation to the Increasing Degeneracy of Human Teeth.. 27
Metallotherapy.................................................... 30
American Academy of Dental Science................................ 35
A New and Original Method of Treating Exposed Pulps............... 36
The Micrococci of Tuberculosis.................................... 37
Metallic-Cap Filling.............................................. 37
Periods of Incubation of the Communicable Diseases................ 40
The Pathology of Epilepsy......................................... 40
Puerperal Infection in the Male................................... 42
The Causes of Death in Artificial Tetanus......................... 42
The Pathology of Daltonism........................................ 43
The Causes and Treatment of Goitre................................ 45
The Freezing Cure................................................. 47
Extirpation of the Larynx......................................... 47
Pyrogallic Acid in Soft Chancre................................... 48

EDITORIAL DEPARTMENT.

Our New Volume.................................................... 49
The Protection of Physicians During Epidemics..................... 49
Hospital Saturday and Sunday...................................... 51
The American Public Health Association............................ 52
Small-Pox Decreasing in New York.................................. 53

CURRENT NEWS AND OPINION.....................................53, 54, 55, 56

REVIEWS AND BIBLIOGRAPHICAL NOTICES...............................57, 58, 59

POPULAR SCIENCE DEPARTMENT.

The Work of Worms................................................. 59
Military Ants of the Amazon....................................... 68
A Plague Among the Violets........................................ 70
Civilization and Decay of the Teeth............................... 71
The Light of the Stars............................................ 71

